# Redirecting linear hydrocarbon metabolism toward polyhydroxyalkanoate biosynthesis

**DOI:** 10.1186/s12934-025-02914-7

**Published:** 2026-01-19

**Authors:** Rocío Palacios-Ferrer, María T. Manoli, Patricia Godoy, Antonio Delgado, Auxiliadora Prieto, Juan L. Ramos

**Affiliations:** 1 Department of Environmental Protection and Biotechnology, Estación Experimental del Zaidín, CSIC, Granada, Spain; 2 Polymer Biotechnology Group, Department of Microbial and Plant Biotechnology, Margarita Salas Center for Biological Research /CIB-CSIC, Madrid, Spain; 3 Andalusian Institute of Earth Sciences, CSIC, Armilla, Granada, Spain

**Keywords:** *Pseudomonas putida*, Alkane monooxygenase, Beta-oxidation pathway, Polyhydroxyalkanoate, ^13^C-isotopic labelling, Linear alkanes, *phaZ*-deficient mutant

## Abstract

This study explores how *Pseudomonas putida* EM2-4 metabolizes linear alkanes after acquiring an integrative and conjugative element (ICE) encoding a novel alkane monooxygenase (AlkB). This AlkB enzyme is a fusion of a hydroxylase and a fatty acid desaturase, exhibiting a narrow substrate range limited to *n*-octane and *n*-decane. The oxidation of these hydrocarbons yields octanoate and decanoate, respectively, which then enter the β-oxidation pathway and the glyoxylate shunt. Under nitrogen-limiting conditions, excess carbon is redirected toward polyhydroxyalkanoate (PHA) synthesis, a phenomenon particularly pronounced in a *phaZ*-deficient mutant unable to depolymeraze PHA, leading to up 75% of cell dry weight. Analysis of the PHAs monomer composition revealed variations with the carbon source. Additionally, ^13^C-isotopic labeling uncovered a minor but unexpected fraction of C10 monomers from C8 substrates, potentially arised from gluconeogenesis followed by the de novo synthesis of fatty acids. Proteomic analyses showed that the carbon source influences the activation of metabolic pathways, correlating with the observed variations in PHAs monomer composition.

**Supplementary Information:**

The online version contains supplementary material available at 10.1186/s12934-025-02914-7.

## Introduction

The industrial revolution, which began in the mid-19th century, brought significant advances for humanity, including improvements in housing, transportation, health care and numerous other areas. However, these developments also led to the overexploitation of natural resources and the synthesis of countless new molecules unknown to nature [1, 2]. These compounds which are commonly referred to as xenobiotics are often recalcitrant, remaining unchanged in the environment or undergoing biotransformation into other chemicals, whose risks are poorly understood.

Short- and medium-chain linear hydrocarbons can be considered as natural molecules because they are produced by certain microbes and algae [3, 4]; however, their current prevalence in the biosphere is mainly the result of the exploitation of fossil fuels. Their ubiquity is largely due to the use of combustion engines and accidental spills in the petrochemical industry (e.g. from petroleum transport ships, or pipelines breaks and leaks) [5], making them dangerous pollutants [6].

Linear hydrocarbons and other anthropogenic pollutants can serve as nutrient sources for microbial growth; however, their degradation as carbon substrates, whilst aiding in pollutant removal, inevitably generates CO_2_ or methane, depending on whether process occurs under aerobic or anaerobic conditions, respectively [7]. A promising alternative is to re-direct these compounds into value-added biochemicals, such as polyhydroxyalkanoates (PHAs) [8, 9], xanthan gums [10] and others [11]. Nonetheless, harnessing microbes for both pollutant C sequestration and biopolymer recovery requires advanced biotechnological strategies. In this context, we developed a strategy enabling *Pseudomonas putida* KT2440 (pWW0)—a non-pathogenic microorganism recognized by the NIH as a safe cloning host [12]—to use *n*-octane as its sole C source. This was achieved by mating *P. putida* KT2440 (pWW0) with a hydrocarbon-degrading community from a fuel-contaminated airport runway wash. Although the community could metabolize *n*-octane, isolating non-pathogenic strains on minimal medium proved difficult [13]. Following the mating, we successfully rescued a KT2440 (pWW0) derivative, designated as *P. putida* EM2-4, that had acquired an integrative and conjugative element (ICE) from the community, which contains a set of *oct* genes responsible for oxidizing *n*-octane to octanoic acid. Genomic analysis revealed a 62-kb DNA ICE (64.86% G + C content, 58 ORFs) assembled from diverse microorganisms [14, 15], including *Burkholderia*,* Delftia*,* Variovorax*, and others [13].

The ICE carries a novel alkane-degradation cluster (*alkB*,* alkJ*,* alkH*,* alkK*) organized differently from the well-characterized *alk* genes of the OCT plasmid of *Pseudomonas putida* (before *P. oleovorans*) GPo1 [16–18]. Unlike the plasmid cluster, the ICE lacked *alkS*,* alkT* and as well as the alkane chemotaxis gene (*alkN)* [16, 19] (Suppl. Figure 1). Furthermore, the ICE-*alk* cluster lacks genes that encodes typical electron donors (a soluble NADH-rubredoxin reductase, and a rubredoxin) [20–22], suggesting reliance on host proteins for AlkB-catalyzed reactions. Functional analyses showed that the fatty acids derived from *n*-alkane are funneled into central metabolism via the glyoxylate shunt [13, 23–26].

In this study, we demonstrate that *P. putida* EM2-4 metabolizes medium-chain alkanes (C8-C10) through a fused alkane monooxygenase while simultaneously producing and storing PHAs in a genetic background deficient in the PhaZ depolymerase. This dual capacity for pollutant degradation and biopolymer production highligts a biotechnological strategy aligned with circular economy principles and the United Nations Sustainability Development Goals (SDGs).

## Materials and methods

### Bacterial strains and growth conditions

*Escherichia coli* BL21 (DE3) strain was utilized for gene expression analysis [27], and *E .coli* DH5α [28] was used for cloning (Suppl. Table 1). *Pseudomonas putida* strains were grown on LB or M9 minimal medium supplemented with different carbon sources [29]. Cultures were incubated at 30 °C with shaking at 200 revolutions per minute (r.p.m.) on an orbital platform. For PHAs analyses, *P. putida* strains were grown in M8-NL (nitrogen-limited) minimal medium containing: 60 g/L NaH_2_PO_4_ × 7H_2_O, 30 g/L K_2_HPO_4_, 1.66 g/L NH_4_Cl, 5 g/L NaCl, 0.52 g/L MgSO_4_ and 2.5 ml of A9 solution [29]. Carbon sources were added at 20 mM for glucose and 15 mM for *n*-octane and other hydrocarbons, these concentrations warrant excess C under N-limiting conditions [29].

To monitor growth, overnight cultures were harvested by centrifugation, washed once with NaCl 0.85% (w/v) and resuspended to turbidity at 660 nm (OD_660_) of 0.1 in 25 mL of M9 minimal medium placed in 100 mL conical flasks. OD_660_ was monitored by using a UV- 1900i UV–vis spectrophotometer (Shimadzu, USA).

### Construction of *P. putida* mutants

Deletion mutants were constructed using the pEMG knockout system, following the method of Martínez-García and de Lorenzo [30], which enables markerless gene replacement via double-strand chromosomal breaks. Primers used (listed in Suppl. Table 2) were designed to amplify the homology DNA-arms flanking the target gene. These DNA-arms were joined by overlapping PCR and cloned into the pEMG vector, flanked by I-SceI restriction sites. Recombinant clones were screened for kanamycin sensitivity on LB agar to confirm plasmid integration. The helper pSW-I-SceI, which encodes the I-SceI endonuclease under an inducible promoter, was electroporated into the cells. Recombinant clones were selected on LB agar supplemented with ampicillin and 3-methylbenzoate (3MB) [31], which induces I-SceI mediated double-strand breaks and stimulates homologous recombination between the flanking regions.

Colonies were screened for kanamycin sensitivity and confirmed by colony PCR screening using primers targeting the flanking regions of the deleted gene. PCR products were sequenced to verify precise deletions (Suppl. Table 1). Finally, plasmid curing was achieved by growing mutants under non-selective conditions and identifying ampicillin-sensitive colonies, ensuring loss of the pSW-I-SceI plasmid.

### Protein expression and identification by mass spectrometry analysis using MALDI-TOF/TOF

*E. coli* BL21 (DE3) bearing the alkane monooxygenase gene cloned in pET28 was grown at 18 °C and 200 r.p.m. overnight. Cells were harvested and resuspended in 1 ml lysis buffer with phenylmethylsulfonyl fluoride (PMSF) and lysed by sonication on ice. Cell debris was removed by centrifugation at 13,000 x *g* for 5 min at room temperature. Proteins were separated on 12% (w/v) SDS-PAGE gels, and bands of interest were excised. Each band was treated with 10 mM dithiothreitol (DTT) during 30 min at 56 °C for reduction, followed by alkylation with 55 mM iodoacetamide during 15 min in the dark. Digestion with trypsin was carried out overnight at 37 °C overnight with 12.5 ng µL^− 1^ sequencing-grade trypsin in 25 mM ammonium bicarbonate (pH 8.5). After digestion, 1 µL of the supernatant was spotted on a MALDI target plate, air-dried, and overlaid with 0.6 µL of α-cyano-4-hydroxy-cinnamic acid matrix (3 mg mL^− 1^ in 50% acetonitrile).

MALDI-TOF MS analyses were conducted using an AB Sciex 4800 Plus Proteomics MALDI-TOF/TOF analyzer, operated in positive reflector mode at 20 kV. Spectra were internally calibrated using trypsin autolysis peptides.

Protein identification was performed using ProteinPilot 4.5 software in conjunction with MASCOT 2.8, querying the UniProt database (https://www.uniprot.org) along with a custom in-house database. The search parameters included: fixed modification of carbamidomethyl cysteine, oxidized methionine as variable along with 80 ppm peptide mass tolerance and missed cleavage allowance (1 trypsin). Proteins with MASCOT scores above the significance threshold (*p* > 0.05) were confidently identified.

### Analysis of octanoate and glucose in culture supernatants

Quantification of octanoate and glucose was carried out via high-performance liquid chromatography (HPLC) using a Shimadzu Prominence series 20 equipped with a Bio-Rad Aminex HPX-87 H column (300 mm x 7.0 mm, particle size 2.2 μm; Hercules, CA, USA), an autosampler (SIL-20ACHT, Shimadzu) and a photodiode array detector (SPD-M20A, Shimadzu). The column was maintained at 30 °C, and 5 mM H_2_SO_4_ was used as the mobile phase at a flow rate of 0.4 mL min^− 1^.

### PHAs analysis

The content and composition of the PHAs were determined by GC/MS after methanolysis [32]. At least three independent biological replicates by duplicate were analyzed; when the standard deviation was > 10% a third duplicate sample was analyzed. About 2 to 5 mg of lyophilized pellets were resuspended in 2 mL methanol containing 15% (v/v) sulphuric acid and 2 mL chloroform with 0.25 mg mL^− 1^ 3MB (internal standard, Sigma-Aldrich, Merck, Germany) [33]. Samples were incubated for 5 h at 105 °C. After cooling, 1 mL of distilled water was added to remove the cell debris. A two-phase extraction process was then carried out to completely remove the water. The organic phase containing the methyl esters of the monomers was analyzed by GC/MS as described [32, 33]. Monomers were unequivocally identified by matching their mass spectra to the standard NIST MS Search v.2.4 spectral database. Under the conditions used, retention times of the monomers were as follows: 3.5 min for C6, 7.2 min for C8, 9.3 min for C9, 12 min for C10, 14.1 min for C11:1; 16.2 min for C12:1; 16.6 min for C12, 20.9 min for C14, and 6.1 min for the internal standard (3MB).

## Results and discussion

### Identification of an oxidoreductase-fused alkane monooxygenase

**Fig. 1:**
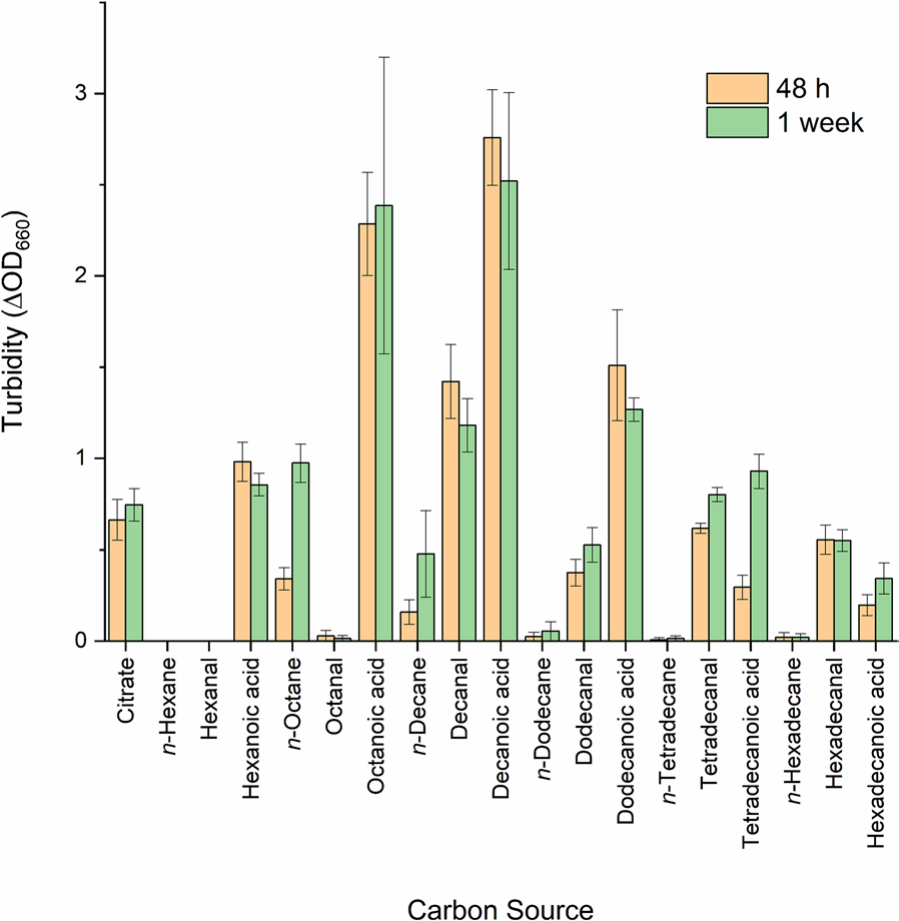
Growth of *Pseudomonas putida* EM2-4 on different linear hydrocarbons and their derivative alcohols, aldehydes, and acids. *P. putida* EM2-4 was grown in M9 minimal medium with citrate as the sole carbon source. After 24 h, 0.1 mL of culture was transferred to 20 mL M9 minimal medium containing the indicated carbon sources at concentrations of 10 mM for hydrocarbons, alcohols, aldehydes, and acids. Culture turbidity was monitored over time at 660 nm (orange bars, 48 h; green bars 1 week). Values are the mean ± standard deviation from at least three independent assays performed in duplicate

We previously reported that *P. putida* EM2-4 can use *n-*octane as a carbon source; however, the full range of *n-*alkanes degraded by this strain have remained unknown. To address this, we tested the ability of *P. putida* EM2-4 to grow on linear hydrocarbons of different chain lengths (Fig. 1). We found that *n*-decane supported growth, whilst linear hexane, dodecane, tetradecane or hexadecane did not. This indicates that the acquired alkane monooxygenase has a relatively narrow substrate specificity. The cultures grown on *n-*octane/*n*-decane reached turbidity values of between 0.6 and 1 at 660 nm with these hydrocarbons, and cell densities of 1 to 3 × 10 ^8^ CFU/ml. Additionally, we observed that the strain could grow on several alcohols (1-decanol, 1-dodecanol, 1-tetradecanol, 1-hexadecanol) and aldehydes (decanal, dodecanal, tetradecanal and hexadecanal), reaching in all cases cell densities of around 5 × 10^8^ CFU/ml. However, no growth occurred with 1-hexanol, hexanal, 1-octanol and octanal (not shown), probably due to their toxicity, as the cells were not viable in the presence of these compounds (cell density dropped by 5 orders of magnitude after addition of 1% of these compounds). In contrast, all tested fatty acids potentially resulting from linear alkane oxidation were indeed utilized as carbon sources. This suggests that the initial oxidation step catalyzed by the alkane monooxygenase is the key limiting factor for growth, reinforcing the conclusion that the enzyme has a narrow substrate specificity.

ORF41 in the acquired ICE element encodes a 754-amino acid protein with homology to fatty acid desaturases/alkane monooxygenases (Suppl. Table 3) [34]. INTERPRO analysis (https://www.ebi.ac.uk/interpro/) for sequence classification and comparison [35] revealed that the protein contains at least two defined domains: one domain spanning residues 1 to 333, with similarity to oxidoreductase proteins, and a second domain, from residue 334 to 754, characteristic of alkane monooxygenases (Suppl. Figure 2-A). These findings suggested that ORF41 could encode a fusion protein of an alkane monooxygenase and a fatty-acid desaturase. BLAST sequence analysis of the two domains separately showed that the half desaturase-like domain shares homology with proteins from *Sphingobium*, *Pseudomonas* and *Tepidicella*, while the AlkB-like domain aligns with sequences from *Pseudomonas* and *Variovorax*.

To verify that this fusion protein was not a sequencing artifact, we re-sequenced the region using Sanger sequencing and specific primers, confirming the presence of the original fusion (Suppl. Figure 2-B). We then cloned the 2.2 kb fragment into the expression vector pET28 and expressed it in *E. coli* BL21. SDS-PAGE revealed that *E. coli* produced a polypeptide of about 83 kDa, consistent with the expected size of the fusion protein (Suppl. Figure 2-D). The band corresponding to this protein was extracted and submitted to peptide mass fingerprinting [36], confirming the authenticity of the expressed protein.

Although fusions between AlkB and desaturases domains have not been previously described, other studies have documented fusions of AlkB with ferredoxin and ferredoxin reductase [37], forming a three-domain complex that only required NADH for activity. These complexes exhibited clear preference for C10-C12 alkanes. Additionally, fusions between AlkB and rubredoxin have been reported [38], effectively linking the electron donor to the alkane monooxygenase. Williams et al. [37], showed that such fusions are widely distributed in nature, and exhibit a substrate profile similar to the non-fused AlkB protein. To further analyze the organization of domains in the fusion protein, we used AlphaFold software (https://alphafold.ebi.ac.uk) to model its potential three-dimensional organization. The model suggested that both “functional” domains folded independently (Suppl. Figure 2-C).

**Fig. 2:**
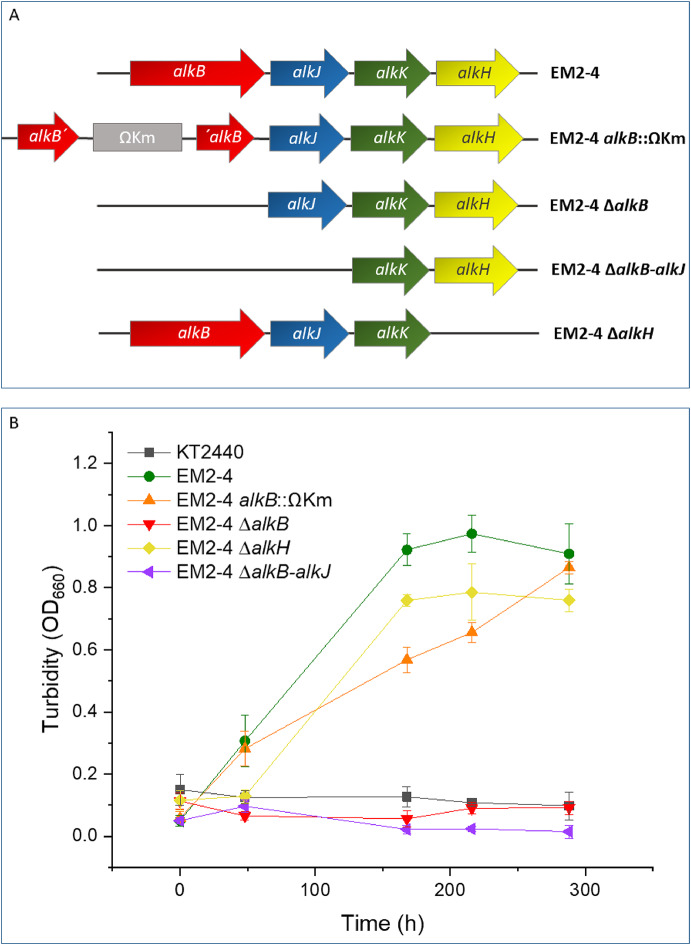
Organization of the *alk* operon in *Pseudomonas putida* EM2-4 and its mutant variants (**A**) and growth on octane (**B**). The top panel shows the gene order and transcriptional direction of the genes in the *alk* operon as described by Duque et al. [13]. For growth assays, strains were grown overnight in M9 minimal medium with citrate, then diluted 50-fold in fresh M9 medium containing 15 mM *n*-octane. Growth was monitored over time. Black square: KT2440 (negative control). Green dot: parental EM2-4. Orange triangle: EM2-4 Ωkm insertion in *alkB* without disrupting hydroxylase activity. Red inverted triangle: *alkB* deletion mutant. Yellow diamond: *alkH* deletion mutant. Blue side triangle: mutant lacking the complete *alkB* and *alkJ* genes. Data represent the mean ± standard deviation of three independent assays

To explore the functional relevance of the fused desaturase/AlkB protein, we generated several mutants of *P. putida* EM2-4 targeting this region by site-directed mutagenesis. In one mutant (EM2-4 *alkB*::ΩKm) a kanamycin resistance cassette was inserted at the junction between the desaturase and the *alkB* domain. In another construction (EM2-4 ∆*alkB*-*alkJ*), the entire fused gene and the adjacent alcohol dehydrogenase were deleted. A third mutant (EM2-4 ∆*alkB*) contained a deletion of only the AlkB-encoding protein. We also generated a knock out mutant of the alcohol dehydrogenase encoded downstream of *alkB* gene (EM2-4 ∆*alkH*). We then tested the growth of the wild-type KT2440, EM2-4, and the different mutants. As expected, all the strains were able to grow on citric acid, but only EM2-4 and the mutant strains that had preserved intact the AlkB domain were able to use *n*-octane as the sole C-source. Mutants which lack the *alkB* gene lost this ability (Fig. 2). These results suggest that the KT2440 genome encodes a native alcohol dehydrogenase capable of oxidizing 1-octanol to octanal, compensating for the loss of the ICE-encoded enzyme.

### Channeling excess C8 carbon source to PHAs in *P. putida* EM2-4

As part of our aim to convert *n-*octane (a known pollutant) into valuable chemicals, we explored its in vivo biotransformation into PHAs. *Pseudomonas putida* produces PHAs as means of carbon storage, primarily under nitrogen-limited conditions when carbon is abundant, though accumulation also takes place at reduced levels under balanced growth [39]. Independently of the carbon source, PHAs synthesis is mediated by the *phaC1* and *phaC2* genes encoding the PHAs polymerases that incorporate (R)-3-hydroxyacyl-CoA monomers derived from fatty-acid catabolism or from the *de novo* synthesis of fatty acids [39–44]. Depolymerization of PHAs is carried out by the *phaZ* gene product, so that the resulting hydroxyalkanoates are channeled into cellular metabolism [41]. Although PHAs synthesis is independent of the C-source, the monomer composition of PHAs is influenced by the specific carbon source used. In fact, *P. putida* (previously *P. oleovorans*) GPo1 thrives on C6–C12 alkanes via the β-oxidation pathway and, under N-limiting conditions, converts the 3-hydroxy fatty-acid intermediates into intracellular PHAs [17, 45]. The resulting monomer composition reflects the alkane source, including the predominant monomer, whose number of carbons corresponds to the alkane chain length, and smaller monomers. When cultivated on *n*-octane, *P. putida* GPo1 synthesizes PHAs containing (R)-3-hydroxyoctanoate and (R)-3-hydroxyhexanoate in a 9:1 ratio [45, 46].

*P. putida* KT2442 strain when grown on glucose under nitrogen-limited conditions also accumulates PHAs, predominantly made up of 3-hydroxydecanoate (around 70%) along with 3-hydroxyhexanoate (approximately 2%), 3-hydroxyoctanoate (about 20%), 3-hydroxydodecanoate, 3-hydroxydodecenoate, 3-hydroxytetradecanoate, and 3-hydroxytetradecenoate (less than 1%) [45]. All these monomers derive from intermediates produced by the *de novo* synthesis of fatty acids. Furthemore, De Eugenio et al. [39] obtained results supporting a model in which PHAs metabolism in *P. putida* KT2442 operates as a continuous cycle. Under carbon-rich conditions, PHAs synthases channel excess carbon into PHAs storage, whilst during carbon limitation, PhaZ releases R-hydroxyalkanoates, which are activated by granule-associated Acyl-CoA synthetase 1 (ACS1) into hydroxyacyl-CoAs. These intermediates are either reincorporated into PHAs or funneled into β-oxidation for energy production, showing the integrated control of polymer synthesis and degradation that sustains cellular homeostasis [39, 47].

**Fig. 3:**
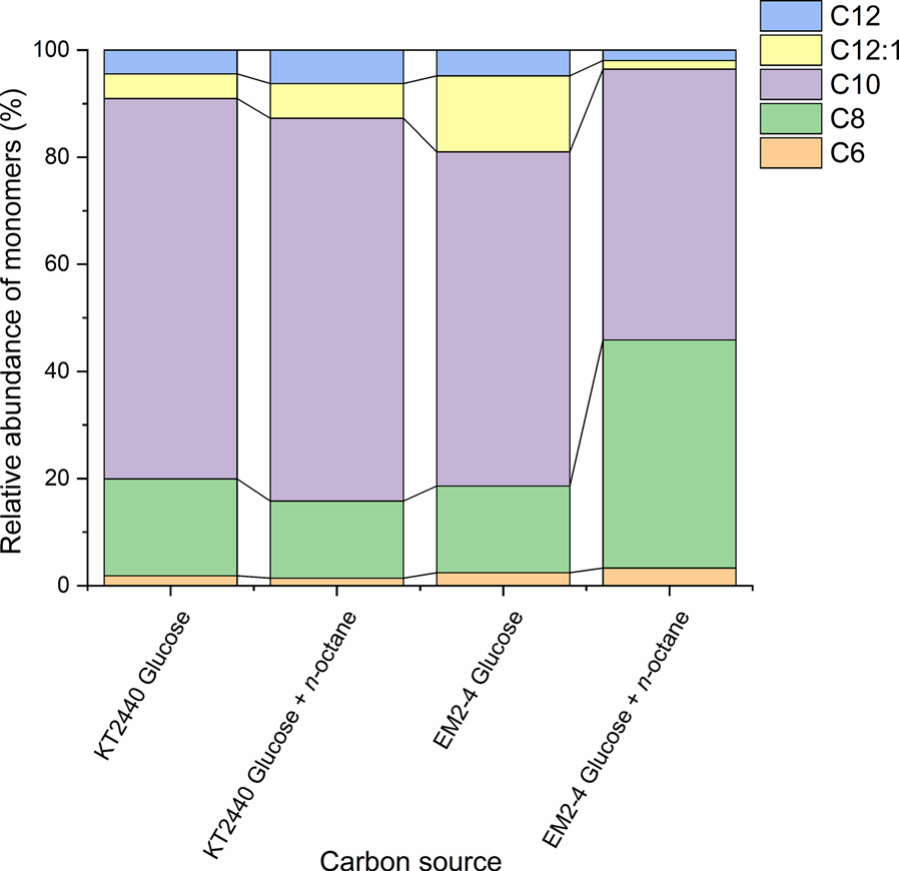
Relative monomer composition in PHA produced by KT2440 and EM2-4 grown in M9 minimal medium with glucose or glucose plus *n-*octane. Cultures were grown for 24 h, PHA was extracted as described in the experimental procedures, and analyzed by GC-MS according to Manoli et al. [33]

Whilst genes for the metabolism of alkanes in the model *P. putida* GPo1 are borne on the OCT plasmid and the expression of *oct* genes is regulated by the *alkS* gene product [16, 21, 48, 49], *P. putida* EM2-4 strain assimilates *n-*octane constitutively, and the genes are located in the chromosome [13]. To investigate the potential production of PHAs by this constitutive *n-*octane consumer *P. putida* EM2-4, we first cultivated *P. putida* KT2440 (pWW0) (the parental strain of EM2-4) and *P. putida* EM2-4 in nitrogen-limited minimal medium with either glucose alone or glucose plus *n-*octane, and determined both PHAs accumulation and its monomeric composition. To be noted that glucose in *P. putida* is assimilated via the Entner-Doudorroff pathway [50], while short- and medium chain linear alkanes are metabolized via the beta-oxidation pathway [16, 24, 26, 49]. After 24 h, up to 30% of the cell dry weight (CDW) consisted of PHA. The monomeric composition of PHAs in *P. putida* KT2440 was consistent with previous findings for *P. putida* KT2442 grown on excess glucose [45], i.e., C6 (2 to 3% of total), C8 (20 to 25% of total), C10 (approximately 70% total), and C12 (8–10% of total, note that we did not detect C14 monomers) (Fig. 3). A similar monomer pattern and ratio was found when KT2440 (pWW0) was grown on glucose plus *n*-octane, agreeing with the inability of KT2440 to assimilate this linear hydrocarbon (Fig. 3). In contrast *P. putida* EM2-4 exhibited a monomer profile similar to KT2440 (pWW0) when grown on glucose alone (Fig. 3); however, in the presence of both glucose and *n-*octane, the proportion of C8 monomers increased significantly (e.g. from 20% to 40%) (Fig. 3). This observation aligns with the findings by Duque et al. [13], which previously showed *P. putida* EM2-4 can simultaneously metabolize glucose and *n*-octane. The increased proportion of C8 monomers probably results from *n*-octane metabolism via the β-oxidation pathway.

When *P. putida* EM2-4 was cultivated on *n*-octane alone, we observed low levels of accumulation of PHAs (e.g. 2% of CDW), probably due to the reduced rate of *n*-octane consumption (4 mg L^− 1^ h^− 1^) compared to 20 mg L^− 1^ h^− 1^ when glucose was present. Moreover, as PHAs levels are influenced by the continuous cycling of synthesis and degradation through the concerted action of PHAs polymerases and PhaZ depolymerase, it is plausible that under growth with *n*-octane alone, PHAs depolymerization is favored due to C limitation [39].

**Fig. 4:**
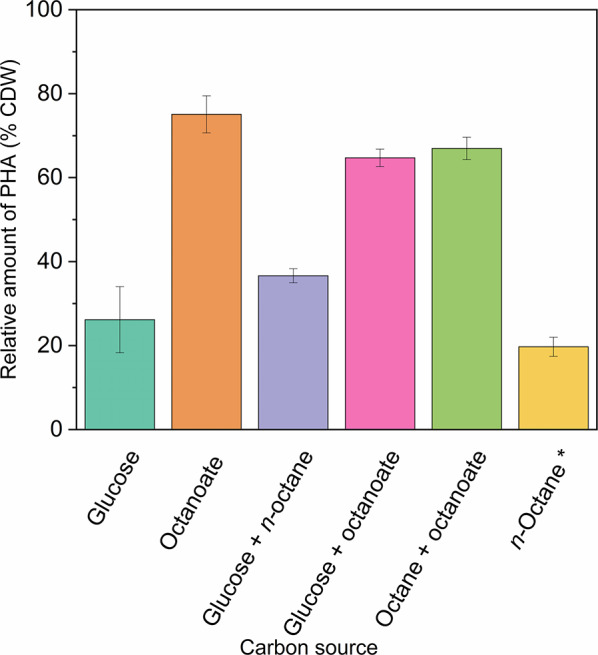
PHA production (% CDW) by an EM2-4 *phaZ* mutant grown with different carbon sources. Cultures were grown for 24 h in M9 minimal medium with the indicated carbon source. Values are the mean ± standard deviation of three independent assays performed in duplicate. *Octane data correspond to accumulation after 21 days, due to deletion of the PHA depolymerase (*phaZ*), accumulated PHA is not degraded

To analyze more precisely the composition of the PHAs produced from *n*-octane, we generated a *phaZ* knock-out mutant of *P. putida* EM2-4 to prevent PHAs degradation. Under nitrogen-limited conditions, the EM2-4-*phaZ* mutant accumulated substantial amounts of PHAs reaching up to 20–25% CDW (Fig. 4). We found that nearly 95% of the PHAs were composed of C8 monomers, with the remainder being C6. Transmission electron microscopy (TEM) analysis confirmed the presence of large intracellular PHAs granules in both the parental *P. putida* EM2-4 and the *phaZ* mutant strains, as expected (Suppl. Figure 3). These findings collectively support that the acquired ICE element enables *n*-octane metabolism via the β-oxidation pathway and that, under nitrogen deficiency PHAs accumulate.

**Fig. 5:**
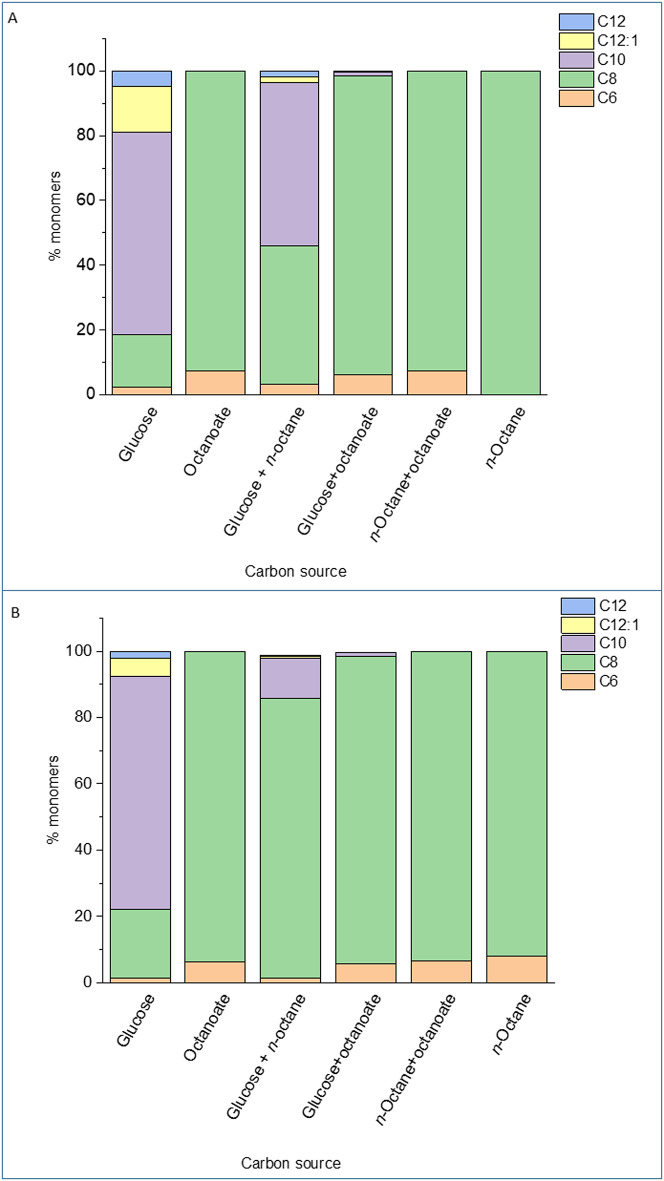
Relative monomer composition in PHA produced by EM2-4 (panel** A**) and its *phaZ* mutant (panel** B**) grown in M9 minimal medium with different carbon sources. Assay conditions are as described in the legend for Fig. 3

**Fig. 6:**
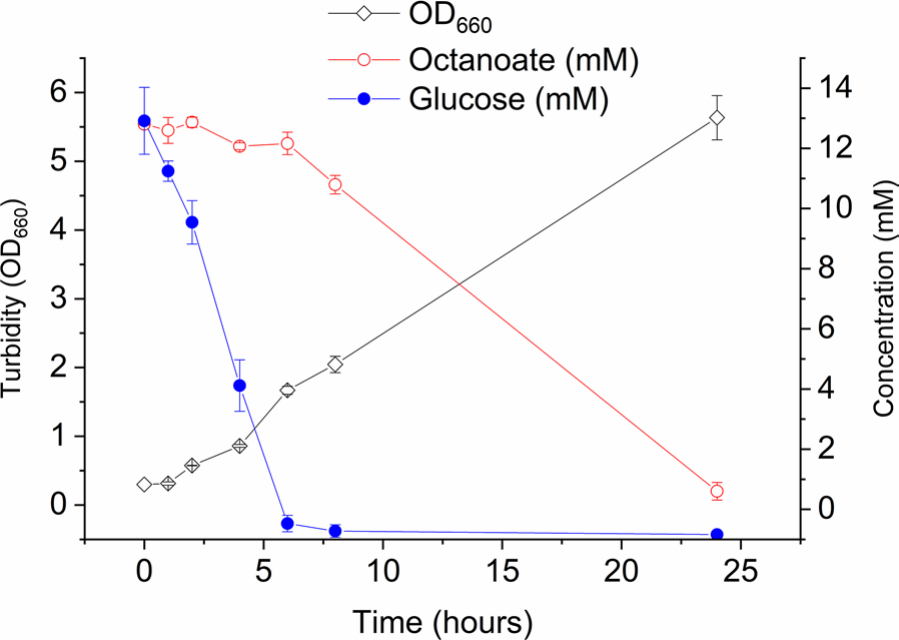
Preferential utilization of glucose over octanoate in *P. putida* EM2-4. Strains were grown in M9 minimal medium with citrate for 24 h, then transferred to fresh M9 medium containing glucose (12 mM) and octanoate (12 mM). Growth was measured as turbidity at 660 nm, and consumption of carbon sources was monitored over time. Values represent the mean ± standard deviation of three independent assays performed in duplicate

**Fig. 7:**
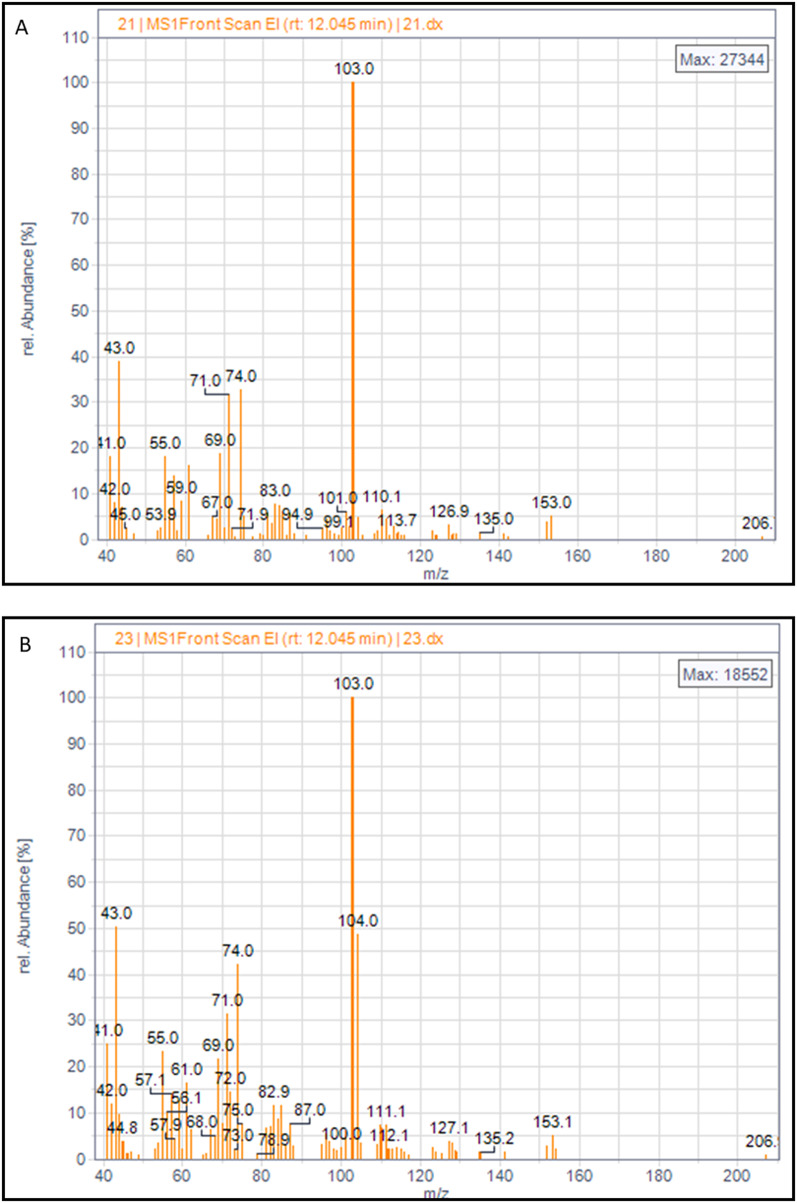
Mass spectra of the C10 monomer in PHA from *P. putida* EM2-4 grown with isotopically labeled carbon sources. Assays used either ^12^C-glucose + ^12^C-octanoate (panel** A**) or ^12^C-glucose + ^13^C-octanoate (panel** B**). Details of isotopic analysis are provided in the experimental procedures

**Fig. 8:**
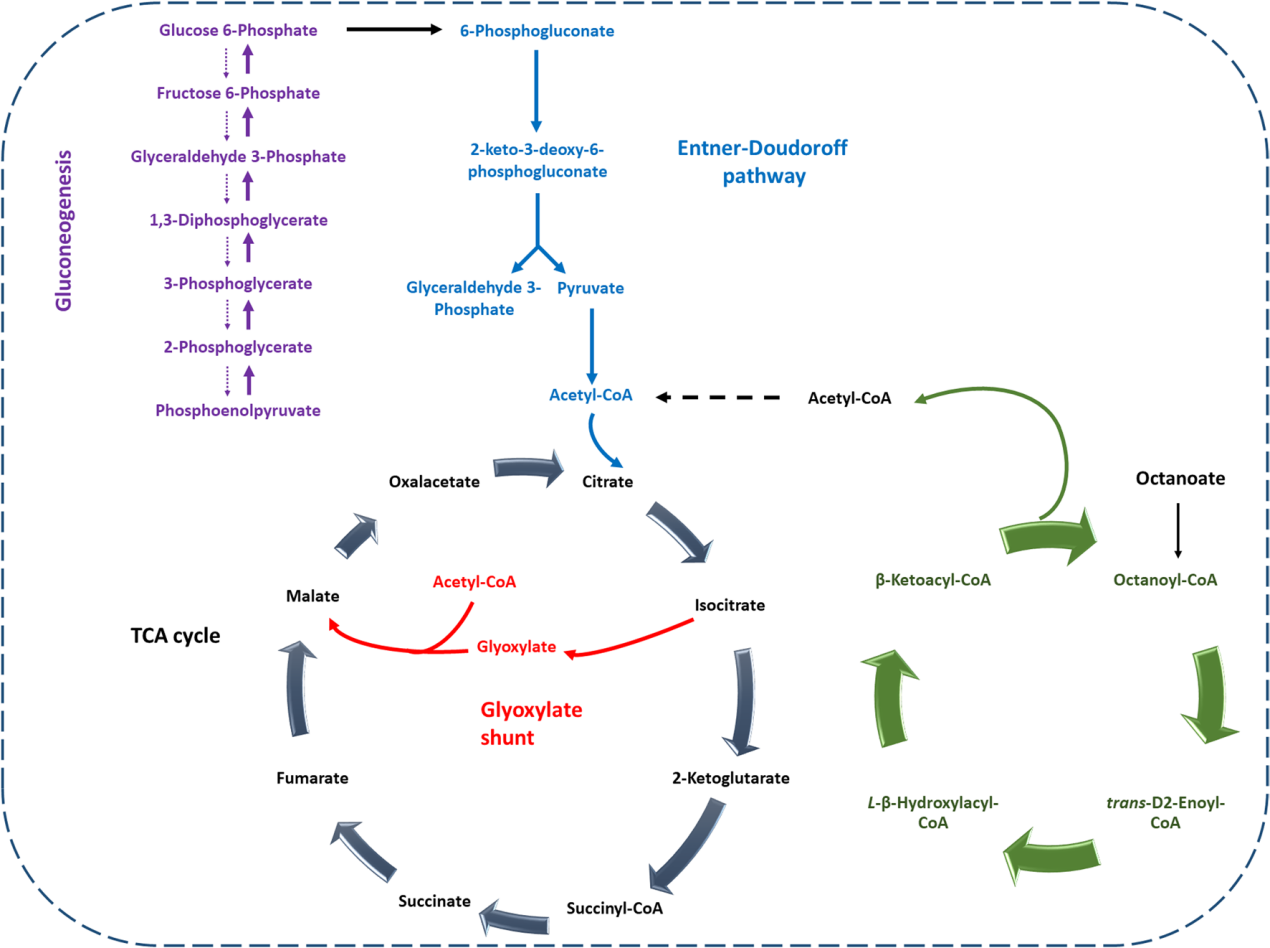
Schematic representation of the interconnection between gluconeogenesis, the β-oxidation cycle, and the glyoxylate shunt in *P. putida* EM2-4

We also analyzed PHAs accumulation in the *phaZ* mutant grown on different C sources (Fig. 5), including glucose, *n*-octane and octanoate, and their combinations. The monomeric composition of PHAs in the mutant cells growing with glucose and octanoate was similar to that of the parental EM2-4. However, an interesting pattern emerged when the *P. putida* EM2-4-*phaZ* mutant was grown on glucose plus *n*-octane or glucose plus octanoate. In cultures with glucose plus *n*-octane, the PHAs was predominantly composed of C8 monomers (> 95% of total) with a notable fraction of C10 (10–13%) and only trace amounts of C6. In contrast, in cultures grown on glucose plus octanoate, the PHAs monomer composition resembled that of cells grown on octanoate alone, with predominance of C8 monomers, low amounts of C6 (less than 5%) and only trace levels of C10. These findings support that octanoate supplied exogenously or produced intracellularly by alkane oxidation is funeled differentially into metabolism.

We also investigated whether gasoline, a complex mixture of linear and aromatic hydrocarbons and paraffins [51], could serve as a carbon source for EM2-4 and its mutant derivative EM2-4 ∆*phaZ*, and whether it could be utilized for PHAs synthesis. Our results revealed that both strains can thrive in the presence of 1 to 3% (v/v) gasoline, reaching cell densities of approximately 2 to 3 × 10^8^ CFU/ml. After 12 days incubation, PHA analysis accounted for only up to 2% of CDW, suggesting that the bioavailable carbon in gasoline could be in excess. Dominguez-Cuevas et al. [52] described that *Pseudomonas putida* exposed to toxic hydrocarbons activate stress responses and efflux pumps, channeling most of their energy toward defense against toxic compounds. It is therefore possible that the low PAHs content in cells exposed to gasoline reflects such stress-related metabolic activity. Further analysis of the monomeric composition of these PHAs revealed that strain EM2-4 mainly produced C12 (47–61%) and C10 (39 to 52%) monomers. In contrast, the *phaZ* mutant also accumulated the C8 monomer, which represented up to 15% of total monomer composition, while C10 and C12 monomers accounted for 57 to 59% and 26 to 34%, respectively. These results suggest a preferential synthesis of PHAs via the *de novo* pathway, likely utilizing C derived from metabolism of aromatic hydrocarbons.

We have also investigated why octanoate is preferentially used for PHAs synthesis over glucose when both compounds were provided simultaneously. To address this we monitored the consumption of these C sources over time and employed ^13^C-labelled isotopes for further insights. We found that glucose was preferentially consumed during exponential growth, while octanoate consumption began only upon entering stationary phase (Fig. 6). To confirm this, we measured CO_2_ evolution over the first three hours of cultivation using either ^13^C-glucose or ^13^C-octanoate. We only detected ^13^CO_2_ when glucose was ^13^C-labelled, indicating that glucose is the preferred carbon source during early growth phase (Suppl. Figure 4). Proteomic analysis further supported this finding. In cells utilizing glucose, all three glucose metabolic pathways were active (Table 1 and raw data deposited in ZENODO: 10.5281/zenodo.17083592), while in cells using octanoate their levels were reduced. When cultures were grown on glucose plus octanoate and when glucose became exhausted, triggering octanoate consumption, proteomic analysis revealed a downregulation of key glucose pathways, particularly the glucose kinase and gluconate pathway, with a less pronounced decrease in the ketogluconate pathway (Table 1). At the same time, we observed induction of key β-oxidation genes and glyoxylate shunt confirming a dynamic shift in the use of the C utilization (Table 1). These findings are consistent with the proposed transition in carbon preference, and explain the pattern of PHAs monomers made by *P. putida* EM2-4.

Finally, we analyzed the isotopic distribution of ^13^C in PHAs when labeled substrates were provided. In GC-MS analysis, the characteristic mass spectrum of unlabeled (^12^C) monomers shows a typical m/z peak of 103, corresponding to the C_4_H_11_O_3_ fragment. Enrichment in ^13^C results in a + 1 shift in mass (m/z of 104) (Fig. 7). When cells were grown on ^13^C-glucose plus ^12^C-octanoate, we only detected the 103 m/z peak, indicating that PHAs were synthesized primarily from octanoate. Conversely, in cultures grown with ^12^C-glucose plus ^13^C-octanoate, we observed both 103 and 104 m/z peaks in the C6 and C8 monomer, and unexpectedly, also a 104-peak in the C10 monomer. We propose that octanoate is metabolized via the β-oxidation pathway and the glyoxylate shunt, with cells generating glucose 6-phosphate (G6P) through gluconeogenesis. Under N-starvation conditions, a small fraction of this glucose-6-phosphate may be metabolized to acetyl-CoA and then directed via the *de novo* synthesis to PHAs (Fig. 8), supporting the notion that PHAs synthesis and degradation are balanced processes in *P. putida.*

**Table 1:** Comparison of proteomic data of EM2-4 growing on glucose or octanoate and transient glucose to octanoate metabolism

Accession	Description	Gene	Abundance ratio (log2)	
			Glu vs. Oct	Glu vs. Glu + Oct
Q88P29	2-Dehydro-3-deoxy-phosphogluconate aldolase	*eda*	2.91	0.31
Q88P38	Mannose/glucose ABC transporter, glucose-binding periplasmic protein	*gtsA*	2.67	1.12
Q88HH8	Epimerase	*kguE*	2.39	1.16
Q88HI0	2-Ketogluconate transporter, putative	*kguT*	2.31	1.49
Q88HH9	2-Ketogluconokinase	*kguK*	2.26	1.14
Q88P35	Mannose/glucose ABC transporter-ATP binding subunit	*gtsD*	2.05	0.69
Q88P30	6-Phosphogluconolactonase	*pgl*	1.94	0.41
Q88P37	Mannose/glucose ABC transporter, permease protein	*gtsB*	1.78	0.7
Q88HE3	D-Gluconate transporter	*gntT*	1.74	− 0.21
Q88P36	Mannose/glucose ABC transporter, permease protein	*gtsC*	1.68	0.6
Q88P31	Glucose-6-phosphate 1-dehydrogenase	*zwfA*	1.67	0.36
Q88P43	Phosphogluconate dehydratase	*edd*	1.56	0.33
Q88HE4	Gluconokinase	*gnuK*	1.53	− 0.35
Q88M85	Long-chain fatty acid transporter		− 1.65	− 0.97
Q88QW6	Acyl-CoA dehydrogenase		− 1.67	− 0.66
Q88QW4	Acyl-CoA dehydrogenase family protein		− 1.69	− 1.42
Q88FI0	Isocitrate lyase	*aceA*	− 1.7	0.15
Q88GG9	3-Hydroxybutyryl-CoA dehydrogenase	*hbd*	− 1.79	− 0.6
Q88L01	3-Ketoacyl-CoA thiolase	*fadA*	− 1.93	− 0.88
Q88GH0	Beta-ketothiolase BktB	*bktB*	− 1.99	− 0.83

## Concluding remarks

This study offers valuable insights into how *Pseudomonas putida* integrates linear alkane metabolism following the acquisition of an integrative and conjugative element (ICE) encoding an unusual alkane monooxygenase. This enzyme is a fusion protein combining a hydroxylase and a fatty acid desaturase, characterized by a narrow substrate specificity, being primarily active on *n*-octane and *n-*decane. Similar substrate range restrictions have been reported for fusion proteins with certain oxidoreductases. Regardless of the evolutionary origin of this unusual enzyme and its limited substrate range, the resulting alcohols /aldehydes/acids derived from *n-*octane and *n-*decane are efficiently funneled through the β-oxidation pathway and the glyoxylate shunt. Under nitrogen-limiting conditions, excess carbon is diverted toward PHAs synthesis, effectively converting pollutants into valuable bioproducts. The PHAs is stably stored as granules in a genetic background deficient in PhaZ. These findings open up new possibilities for manipulating culture conditions to optimize the bioconversion of waste streams into high-value compounds.

## Supplementary Information

Below is the link to the electronic supplementary material.

Supplementary Material 1

## Data Availability

Raw proteomic data are available at: doi: 10.5281/zenodo.17083592),

## Abbreviations

3MB: 3-Methylbenzoate
CDW: Cell Dry Weight
DTT: Dithiothreitol
GC-MS: Gas Chromatography–Mass Spectrometry
HPLC: High-Performance Liquid Chromatography
ICE: Integrative and Conjugative Element
PCR: Polymerase Chain Reaction
PHA: Polyhydroxyalkanoates
PMSF: Phenylmethylsulfonyl fluoride
TEM: Transmission Electron Microscopy
UN-SDG: United Nations Sustainable Development Goals
