# Supplementary material for: Redirecting linear hydrocarbon metabolism toward polyhydroxyalkanoate biosynthesis
Source: Microb Cell Fact. 2026 Jan 19;25:45. doi: 10.1186/s12934-025-02914-7 (PMC12903215; doi:10.1186/s12934-025-02914-7)
Supplement: Supplementary file 1 — Supplementary Material 1 [file 12934_2025_2914_MOESM1_ESM.docx]

# Supplementary material

# Redirecting linear hydrocarbon metabolism toward polyhydroxyalkanoate biosynthesis

Rocío Palacios-Ferrer^1^, María T. Manoli^2^, Patricia Godoy,^1^ Antonio Delgado^3^, Auxiliadora Prieto^2^ and Juan L. Ramos^1*^

^1^ Department of Environmental Protection and Biotechnology, Estación Experimental del Zaidín, CSIC, Granada, Spain

^2^Polymer Biotechnology Group, Department of Microbial and Plant Biotechnology, Margarita Salas Center for Biological Research /CIB-CSIC), Madrid, Spain

^3^Andalusian Institute of Earth Sciences, CSIC, Armilla, Granada, Spain

*Corresponding author:

Juan L. Ramos

Phone: +34958439153

ORCID: 0000-0002-8731-7435

**Supplementary material**

**Material and methods**

### Transmission electronic microscopy (TEM)

Bacterial suspensions were fixed with 2.5% (v/v) glutaraldehyde in 0.1M cacodylate buffer, pH 7.4, for 24h at 4°C. Post-fixation was done with 1% (w/v) osmium tetroxide for 1 h at room temperature and then dehydrated with an ethanol gradient of 50%, 70%, 90%, and 100% (x3), 20 minutes at each step. Critical point drying was performed with CO_2_ using a LEICA EM CPD 300 dryer. Finally, samples were coated with carbon in an EMITECH K975X and imaged in a Transmission Electron Microscope TEM Libra 120 plus with LaB6 (Lanthanum Hexaboride) filament operating at 120 KV (Kilovolts) accelerating voltage, column energy filter for EFTEM imaging and analysis by Electron Energy Lose Spectroscopy (EELS).

**Proteomics**

Proteomics analysis were carried out at the CNB-CSIC proteomic facility Madrid, Spain (http:// proteo. cnb. csic. es/ prote omical) using the iTRAQ protocol (Cuenca et al., 2016; Molina et al., 2019). Cultures were prepared as described for the transcriptomics assays. Cells were harvested by centrifugation (5000 x *g* for 10 minutes at 4 ºC), washed twice with M9 medium and stored at -80 ºC. For protein extraction, bacterial cell pellets were resuspended in lysis buffer containing 5% (w/v) sodium dodecyl sulfate (SDS; Sigma-Aldrich) and 100 mM triethylammonium bicarbonate (TEAB; Thermo Fisher Scientific). Cell disruption and homogenization were performed by micro-tip probe sonication for 1 min using a UP50H ultrasonic homogenizer (Hielscher Ultrasonics). Lysates were centrifuged at 21,000 × *g* for 5 min at 4 °C, and the supernatant containing solubilized proteins was collected for further processing. A portion of the extract was subjected to protein precipitation using the methanol/chloroform method and resolubilized in a denaturing buffer composed of 7 M urea, 2 M thiourea, and 100 mM TEAB. Protein disulfide bonds were reduced and alkylated at 37 °C for 60 min in the presence of 5 mM tris(2-carboxyethyl)phosphine (TCEP) and 10 mM chloroacetamide (CAA). Protein concentrations were determined using the Pierce™ 660 nm Protein Assay (Thermo Fisher Scientific).

Aliquots containing 150 μg of protein were diluted 1:5 in 100 mM TEAB and digested overnight at 37 °C with continuous shaking using MS-grade trypsin (Thermo Fisher Scientific) at an enzyme-to-substrate ratio of 1:20 (w/w).

Peptide samples were labelled with the TMT 18plex™ Isobaric Label Reagent Set (Thermo Fisher Scientific, Rockford, IL, USA) following a protocol modified by the manufacturer. Labelling was conducted in 85% 200 mM EPPS buffer (pH 8.5) and 15% anhydrous acetonitrile. Reactions were incubated at 25 °C for 2 h and subsequently quenched with hydroxylamine to a final concentration of 0.3% (w/v) for 15 min. Labelled peptides were pooled, vacuum-dried, and desalted using Sep-Pak C18 cartridges (Waters). Three biological replicates were analyzed per experimental condition.

Peptide mixtures were desalted using StageTips packed with Empore™ C18 material (Sigma-Aldrich). Peptide concentration was quantified with the Qubit™ Fluorometric Quantitation System (Thermo Fisher Scientific). One microgram of each sample was subjected to one-dimensional nanoLC–ESI–MS/MS analysis using an Ultimate 3000 nano-HPLC system (Thermo Fisher Scientific) coupled to an Orbitrap Exploris 240 mass spectrometer (Thermo Fisher Scientific). Peptides were separated on a microPAC™ C18 column (PharmaFluidics) at 45 °C using a 120 min linear gradient from 2% to 95% acetonitrile (0.1% formic acid) at a flow rate of 250 nL/min. The injection volume was 5µL.

Mass spectra were acquired in data-dependent acquisition (DDA) mode with full MS scans from m/z 350 to 1200 at a resolution of 60,000 (m/z 200), AGC target of 300%, and maximum injection time of 40 ms. The top 20 most intense precursor ions were selected for fragmentation by higher-energy collisional dissociation (HCD) with a collision energy of 34. MS/MS scans were acquired at a resolution of 45,000, with an AGC target of 200% and a maximum injection time of 120 ms. Precursor ions with charge states of +1, unassigned, or ≥+6 were excluded. Dynamic exclusion was set to 45 s.

Raw data were processed with Proteome Discoverer v2.5 (Thermo Fisher Scientific). MS/MS spectra were searched using Mascot (v2.7.0 and v2.4.0), MsFragger (v3.1.1), and Sequest HT search engines. The following modifications were considered: fixed carbamidomethylation of cysteine (+57.021 Da), variable oxidation of methionine (+15.995 Da), pyro-glutamate formation from glutamine (−17.027 Da), and TMT tags on peptide N-termini and lysine residues (+229.163 Da). Trypsin specificity was used, allowing up to two missed cleavages. The precursor and fragment mass tolerances were set to 10 ppm and 0.02 Da, respectively. The false discovery rate (FDR) was controlled at 1% for peptide spectral matches (PSMs), peptides, and proteins.

Protein quantification was based on reporter ion intensities summed across all identified peptides. Peptide group abundances were aggregated per sample and normalized to the maximum total signal across all runs to assess differential protein expression.

Peptide identification was restricted to those with a *q*-value below 0.05. Proteins were considered validated if supported by at least two distinct peptides. The false discovery rate (FDR) for protein identification was estimated to be below 1% using a reversed decoy database approach. Proteins with an abundance change of ≥1.5-fold (log₂ fold change) with a *q*-value ≤0.05 were considered differentially abundant.

**Supplementary figures**


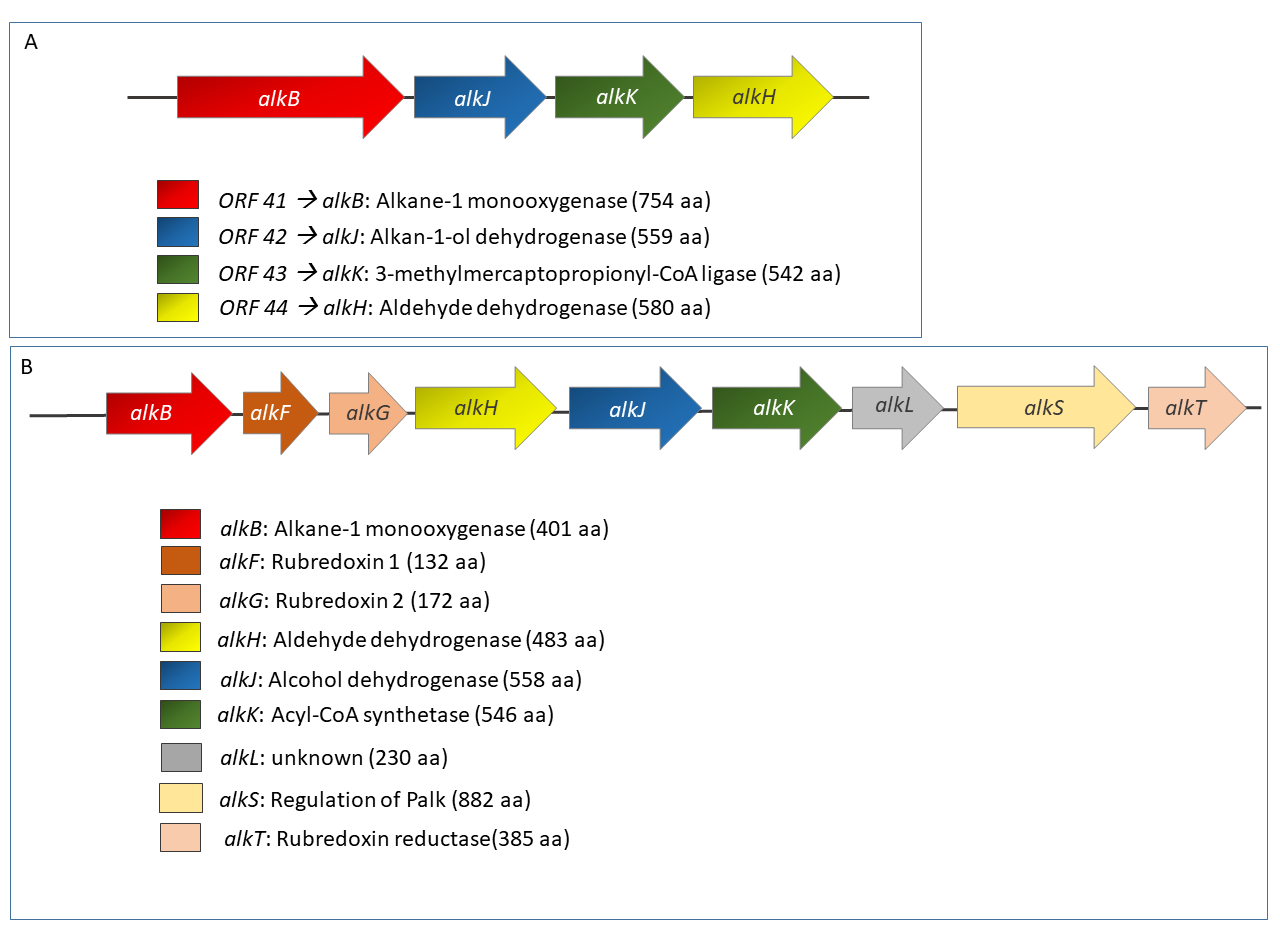


**Supplementary Figure 1.** Comparative figure ICE and OCT plasmid. Genetic organization of the *alk* cluster in the ICE integrated in the EM2-4 chromosome (panel A; Duque et al., 2022) and in the OCT plasmid (panel B; [49]) Arrows indicate transcription direction. Gene product, and predicted size in amino acids are shown below the schematic representation of genes.


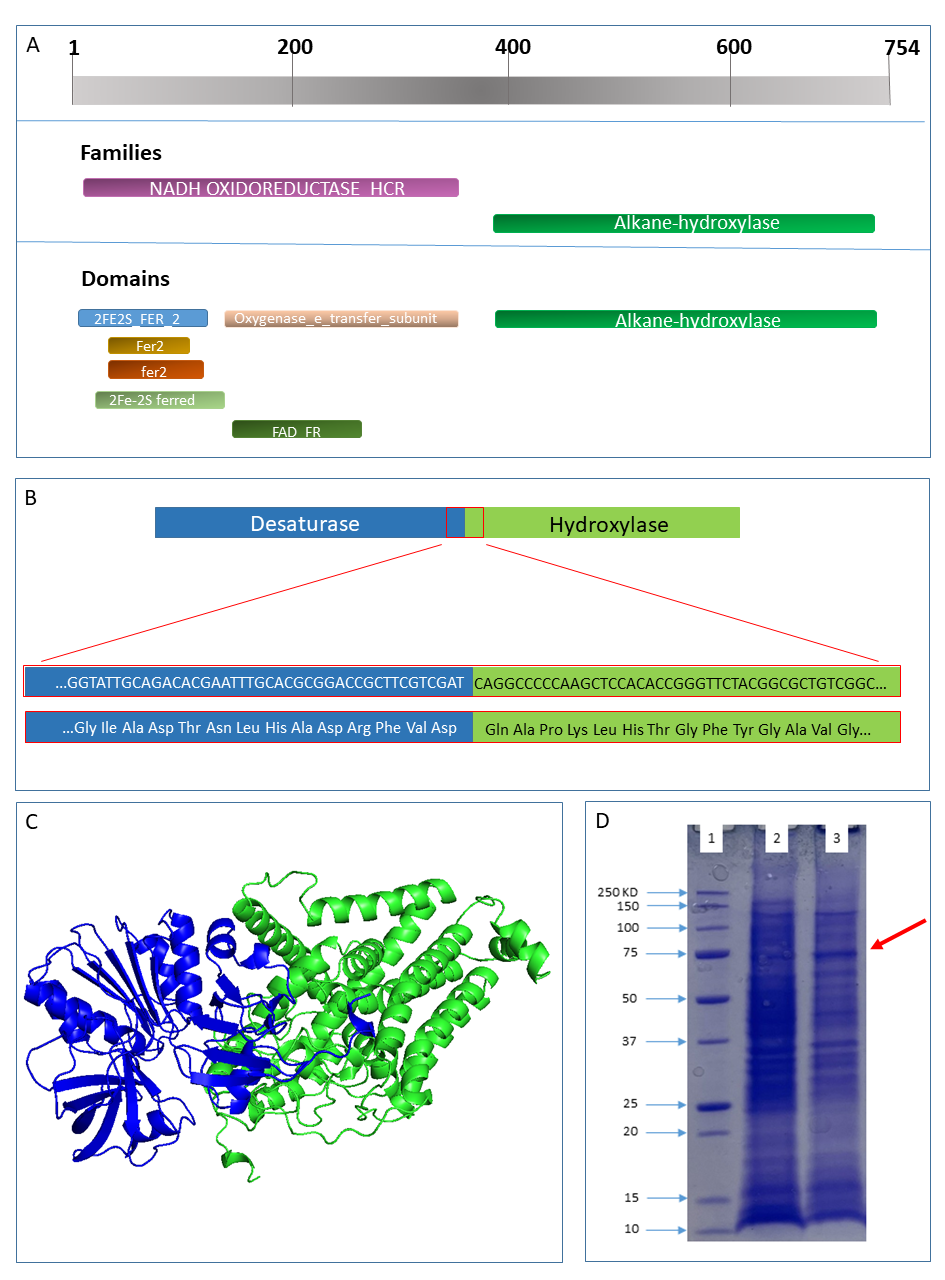


**Supplementary Figure 2.** Analysis of the AlkB protein encoded by the chromosome of *P. putida* EM2-4. A) INTERPRO analysis of the AlkB protein from EM2-4 showing functional domains. B) Nucleotide and protein sequence detail of the fusion between alkane hydroxylase and fatty acid desaturase domains, confirming the validity of the original genome sequencing. C) AlphaFold model of EM2-4 AlkB, indicating potential independent folding of the desaturase (blue ribbons) and hydroxylase (green ribbons) domains. D) Identification of AlkB fusion protein expressed in *E. coli*. Protein extracts from *E. coli* BL21 carrying the EM2-4 *alkB* gene were analyzed by SDS-PAGE (12% w/v). Lane 1: molecular weight markers. Lane 2: non-induced culture. Lane 3: culture induced with 0.5 mM IPTG at 18 °C for 12 h. The AlkB protein band is indicated by an arrow and it was excised for analysis.


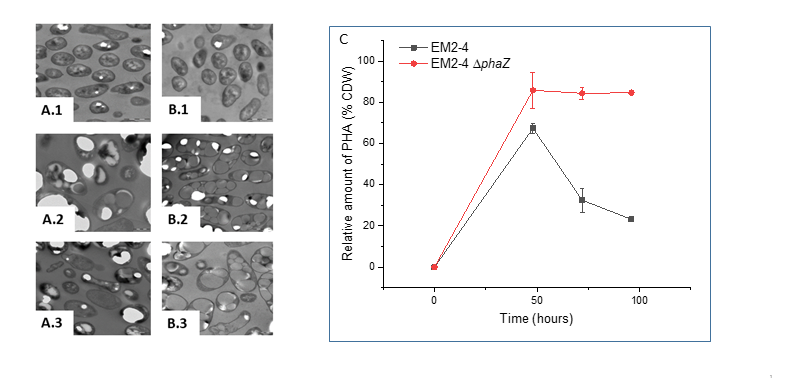


**Supplementary Figure 3.** Dynamics of PHA accumulation in *P. putida* EM2-4 (panel A) and EM2-4 *phaZ* mutant (panel B) growing on M9 with 10 mM octanoate. Panel C: PHA production (% CDW) by the parental EM2-4 and EM2-4 *phaZ* mutant analyzed by GC-MS.





**Supplementary Figure 4.** Early evolution of ^13^CO₂ in cultures of *P. putida* EM2-4 grown for 3 h with glucose and ^13^C-labeled octanoate as indicated. Negative control used ^12^C-glucose and ^12^C-octanoate. Values are the mean of at least four independent assays.

| Strain | Genotype/Relevant characteristics | Reference |
| --- | --- | --- |
| ***Pseudomonas putida*** |  |  |
| KT2440 | pWW0, Rif^R^, Cm^R^, OCT^-^, Citrate^+^ | [12] |
| EM2-4 | KT22440 derivative, pWW0, Rif^R^, Cm^R^, OCT^+^, Citrate^+^ | [13] |
| EM2-4 *alkB*::ΩKm | EM2-4 mutant generated by insertional inactivation of *alkB*, Km^R^ | This work |
| EM2-4 ∆*alkB* | EM2-4 derivative, Δ*alkB* cluster deletion mutant, Km^R^ | This work |
| EM2-4 ∆*alkB-alkJ* | EM2-4 derivative, Δ*alkB-alkJ* cluster deletion mutant, Km^R^ | This work |
| EM2-4 ∆*alkH* | EM2-4 derivative, Δ*alkH-alkJ* cluster deletion mutant, Km^R^ | This work |
| EM2-4 ∆phaZ | EM2-4 derivative, Δpha cluster deletion mutant, Km^R^ | This work |
| ***Escherichia coli*** |  |  |
| DH5α | *Cloning host: F-λ-*endA1 glnX44*(AS)*thiE1 recA1 relA1 spoT1 gyrA96*(NalR)*rfbC1 deoR nupG*Φ80(*lacZ*Δ*M15*) Δ(*argF-lac*)*U169 hsdR17*(*rK*–*mK*+)* | [28] |
| BL21 | Protein expression host: *fhuA2 [lon] ompT gal (λ DE3) [dcm] ∆hsdS λ DE3 = λ sBamHIo ∆EcoRI-B int::(lacI::PlacUV5::T7 gene1) i21 ∆nin5* | [27] |
| CC118 λpir | Cloning host: *araD139* Δ*(ara-leu)7697* Δ*lacX74 galE galK phoA20 thi− 1 rpsE rpoB*(Rif^R^) *argE*(Am) *recA1*, λpir lysogen | [30] |
|  |  |  |
|  |  |  |

**Supplementary table 1.** Strains used in this study.

**Supplementary table 2**

| **Oligonucleotide** | **Sequence (5´-3´)** | **Utility** |
| --- | --- | --- |
| pEMG-Fw | CCATTCAGGCTGCGCAACTGTTG | Deletion mutants |
| pEMG-Rv | CTTTACACTTTATGCTTCCGGC | Deletion mutants |
| pSW-Fw | GGACGCTTCGCTGAAAACTA | pSW curation |
| pSW-Rv | AACGTCGTGACTGGGAAAAC | pSW curation |
| Up-*alkB*-Fw | CGGAATTCAATTGGCGGAAGCCGTTCTA | *alkB* deletion |
| Up-*alkB*-Rv | GGCTGAGGTACCGTGTTCAAAGATGATCTTGCCGATGCGT | *alkB* deletion |
| Dw-*alkB*-Fw | TTGAACACGGTACCTCAGCC | *alkB* deletion |
| Dw-*alkB*-Rv | GCGATGAGACTGGAGACGAGAGATGATCTTGCCGATGCGT | *alkB* deletion |
| Dw-*alkB*-*alkJ*-Fw | CTCGTCTCCAGTCTCATCGC | *alkB-alkJ* deletion |
| Dw-*alkB*-*alkJ*-Rv | CGGGATCCCCGGAGGTGTAACACAACGA | *alkB-alkJ* deletion |
| Up-*phaZ*-Fw | CGGGGTACCGCATCACTTGTACCGCACTG | *phaZ* deletion |
| Up-*phaZ*-Rv | GCAACACTCCCTCGTCTGATCACCAGGAGTCTGCATGCTT | *phaZ* deletion |
| Dw-*phaZ*-Fw | ATCAGACGAGGGAGTGTTGC | *phaZ* deletion |
| Dw-*phaZ*-Rv | CGGGATCCCGTTGCGAAATACCACTGCG | *phaZ* deletion |
| Up-*alkJ*-Fw | CGGAATTCCGCTGTTCGGAAAATCGCTGCTGGAAATCG | *alkJ* deletion |
| Up-*alkJ*-Rv | CTATTGGTATGCAAGAAGTAATACACCAGATATTCATAAAATCCCACTGCGGAGCCGAGGT | *alkJ* deletion |
| Dw-*alkJ-*Fw | ATCTGGTGTATTACTTCTTGCATACCAATAG | *alkJ* deletion |
| Dw-*alkJ*-Rv | CGGGATCCGGGCAGAAAGGTCCGGTCGATGAACAGCAC | *alkJ* deletion |

Supplementary table 2. Oligonucleotides used for deletion mutants construction

| **Description** | **Scientific Name** | **Query cover** | **Percent intent** | ***E*‐value** | **Accesion** |
| --- | --- | --- | --- | --- | --- |
| MULTISPECIES: fatty acid desaturase [*Pseudomonadota*] | *Pseudomonadota* | 100% | 100.00% | 0.0 | WP_100441173.1 |
| Fatty acid desaturase [*Tepidicella baoligensis]* | *Tepidicella baoligensis* | 100% | 94.95% | 0.0 | WP_180683617.1 |
| Fatty acid desaturase [*Sphingobium aquiterrae*] | *Sphingobium aquiterrae* | 100% | 94.15% | 0.0 | WP_336962432.1 |
| MULTISPECIES: fatty acid desaturase [*Pseudomonadota*] | *Pseudomonadota* | 100% | 94.02% | 0.0 | WP_024100076.1 |
| fatty acid desaturase [*Pseudoxanthomonas mexicana*] | *Pseudoxanthomonas mexicana* | 100% | 93.88% | 0.0 | WP_162109262.1 |
| MULTISPECIES: fatty acid desaturase [*Burkholderia*] | *Burkholderia* | 100% | 93.22% | 0.0 | WP_080381189.1 |
| fatty acid desaturase [*Hydrogenophaga sp.*] | *Hydrogenophaga sp.* | 100% | 86.67% | 0.0 | WP_291064615.1 |
| MULTISPECIES: fatty acid desaturase [*Burkholderiales*] | *Burkholderiales* | 100% | 80.43% | 0.0 | WP_011797842.1 |
| 2Fe-2S iron-sulfur cluster-binding protein [*Pseudomonas aeruginosa*] | *Pseudomonas aeruginosa* | 83% | 94.42% | 0.0 | WP_237043526.1 |
| fatty acid desaturase [*Rugamonas apoptosis*] | *Rugamonas apoptosis* | 100% | 76.96% | 0.0 | WP_182156688.1 |
| fatty acid desaturase [*Pseudomonas extremaustralis*] | *Pseudomonas extremaustralis* | 100% | 74.57% | 0.0 | WP_150294357.1 |
| fatty acid desaturase [*Burkholderia sp.*] | *Burkholderia sp.* | 99% | 71.33% | 0.0 | WP_292237936.1 |
| fatty acid desaturase [*Pseudomonas aeruginosa*] | *Pseudomonas aeruginosa* | 77% | 93.07% | 0.0 | MCS8035728.1 |
| fatty acid desaturase [*Burkholderia cenocepacia*] | *Burkholderia cenocepacia* | 71% | 91.98% | 0.0 | WP_230391020.1 |

Supplementary table 3. BLAST analysis of AlkM in EM2-4.
